# Personal Computer-Based Visual Field Testing as an Alternative to Standard Automated Perimetry

**DOI:** 10.7759/cureus.32094

**Published:** 2022-12-01

**Authors:** Muhammad A Khizer, Taimoor A Khan, Umar Ijaz, Summaya Khan, Abdul K Rehmatullah, Izza Zahid, Hira G Shah, Muhammad A Zahid, Haroon Sarfaraz, Nawal Khurshid

**Affiliations:** 1 Ophthalmology, National University of Medical Sciences, Rawalpindi, PAK; 2 Ophthalmology, Armed Forces Institute of Ophthalmology, Rawalpindi, PAK; 3 Ophthalmology, Combined Military Hospital, Quetta, PAK; 4 Ophthalmology, Ohud Hospital,, Madinah-al-Munawarah, SAU; 5 Medical Student, James Cook University, Townsville, AUS; 6 Ophthalmology, Alshifa Trust Eye Hospital, Rawalpindi, PAK; 7 Ophthalmology, Monash Health, Clayton, AUS; 8 Ophthalmology, Wah Medical College, Wah Cantt, PAK; 9 Otolaryngology, Pakistan Institute of Medical Sciences, Islamabad, PAK

**Keywords:** neuro-visual rehabilitation, visual field loss, visual field defect, visual field analysis, open angle glaucoma, glaucoma diagnosis and management, glaucoma practice, primary open angle glaucoma

## Abstract

Introduction

Standard automated perimetry (SAP) is the gold standard of visual field assessment in patients with neuro-ophthalmic conditions. Glaucoma is a progressive optic neuropathy characterized by damage to the ganglion cell complex with corresponding visual field defects and intraocular pressure (IOP) being the only modifiable ocular risk factor. Recent advances in technology have paved the way for remote screening and monitoring of visual field defects with the aid of a computer or tablet-based software. One such personal computer (PC)-based software is ‘Specvis’, which has shown promising reliability as compared to SAP. The primary objective of this study was to compare Specvis and Humphrey Field Analyzer (HFA) visual field reports in the graphical domain while secondary objectives were to estimate the ease of use of Specvis in comparison to HFA and comparison of test duration between Specvis and HFA.

Materials and methods

This was a cross-sectional validation study performed at a tertiary care ophthalmology institute in Rawalpindi, Pakistan. Subjects presenting to the outpatient department were recruited based on consecutive sampling technique and were divided into healthy and diseased groups. Basic data collection instrument after informed consent was filled with demographic data, ophthalmic data, disease condition, and attached with analysis reports of both HFA and Specvis for assessment by three senior ophthalmology consultants independently.

A total of 218 eyes of 109 subjects were included in this pilot study. SAP was done on the VF 30-2 program using HFA 3. The same patient then performed the visual field assessment on a PC with Specvis installed and settings adjusted to match the VF 30-2 program of HFA as closely as possible. Visual fields of a subject obtained from HFA and Specvis were then coupled and sent to three different senior ophthalmologists. The assessment was done by comparing the greyscale visual field printouts in the graphical domain and scored based on a 5-point Likert scale which were then analyzed for inter-observer reliability. After each test, all subjects were asked to rate the difficulty level of performing the test on HFA and Specvis based on a 5-point Likert scale. The duration of the test performed on HFA and Specvis was also noted for comparison.

Results

We observed male preponderance in our study participants (n=128, 58.72%). The majority of the participants were non-diseased (n=170, 77.98%) while advanced glaucoma was the commonest disease in the diseased group (n = 22, 10.09%). The mean age of the participants was 40.71 (SD=15.24). The observations for the HFA test duration had an average of 213.33 seconds (SD=33.49, Min=174.00, Max=314.00) while the Specvis test duration had an average of 267.36 seconds (SD=35.98, Min=228.00, Max=370.00). A significant positive correlation was observed between score 1, score 2, and score 3 given by the three ophthalmologists. A significant negative correlation was observed between ease of using HFA and age, with a correlation of -.28. A significant negative correlation was also observed between ease of using Specvis and age.

Conclusion

Specvis, a computer-based free open-source software used in our study, can give promising results in diagnosing as well as monitoring the progression of visual field defects. It can act as a significantly cost-effective and readily available bridge between visual field examination by confrontation method and SAP.

## Introduction

Standard automated perimetry (SAP) remains the standard of visual field assessment despite many advances in ophthalmic imaging [1]. It is used in various ophthalmic conditions for both diagnoses as well as long-term follow-up. Many of these neuro-ophthalmic conditions are a major cause of partial and/or complete blindness. Of these conditions, glaucoma and neurological disorders causing visual field impairment constitute a major burden and are amenable to treatment if identified early in the disease process [2-4].

It is estimated that about 90% of global blindness is present in developing countries with glaucoma being only second to cataract as a cause of blindness. As a continent, Asia has a disproportionately high prevalence of glaucoma, where it is undiagnosed in nine out of 10 cases [4-6]. A major obstacle to the diagnosis and treatment is the high socio-economic burden associated with running glaucoma screening programs as well as the high costs of equipment used in diagnosing the condition, especially in developing countries [7,8].

Advances in technology and artificial intelligence have now provided the ability to use commonly available devices (personal computers, tablets, mobile phones, etc.) as screening and monitoring devices for conditions such as glaucoma [9,10]. Usage of such readily available devices could significantly reduce the burden on health services in aspects of both human resources and cost-effectiveness, especially in developing countries where health services are already overburdened. Of these novel ideas, the concept of usage of a personal computer (PC) screen for automated perimetry and as a screening device for visual field defects was given by Dzwiniel et al. after an internal validation study in the form of a free and open-source software called Specvis, having the capability to run on almost all types of popular PCs irrespective of its manufacturer or operating system. Although the internal validation revealed that the software can be used as a reliable visual field examination tool, the study was limited by a small sample size including only four glaucoma patients, and comparison to only Medmont M700 Automated Static Perimeter [11].

Based on initial equipment cost estimates, a new PC can be bought for about 550 USD, which is at least a 25-fold decrease in initial cost as compared to Humphrey Field Analyzer (HFA) 3, the SAP machine used in our study [12-14]. This price can go even significantly lower if an older pre-owned PC is bought, which should be nearly equally efficient as a new one because the software is designed to run on a broad range of PCs, newer and older ones alike. Being able to perform automated perimetry on a PC that is reliable as screening as well as monitoring tool would therefore be a very cost-effective and valuable asset, especially in developing countries where SAP devices are available only in tertiary care centers primarily because of their high initial and running costs along with the requirement of trained staff for its use and maintenance [15,16]. Conversely, PCs are affordable, easy to use, and require only an additional initial one-time setting for their use with the software [11]. 

At the time of this study, no external validation study on the software’s capabilities has been published in the literature. This study aims to assess the capabilities of the software on a larger study group as well as to compare the results to another popular SAP device, the HFA. The primary objective of this study was to compare Specvis and HFA visual field reports in the graphical domain while secondary objectives were to estimate the ease of use of Specvis in comparison to HFA and test duration comparison between Specvis and HFA.

## Materials and methods

This was a cross-sectional validation study performed at a tertiary care ophthalmology institute, Armed Forces Institute of Ophthalmology (AFIO) Rawalpindi between February 2021 and September 2022. Institutional ethical review committee approval was obtained prior to the conduction of the study (approval 213/ERC/AFIO). Subjects presenting to the outpatient department were recruited based on consecutive sampling technique and were divided into healthy and diseased groups. 

The inclusion criteria were as follows: Patients with documented visual field defects on SAP (diseased group), individuals with no visual field defect on SAP (healthy group), best corrected visual acuity of at least 6/15 in the tested eye, and age limit between 15 -70 years. The exclusion criteria were as follows: subject unable to perform or understand the usage of Specvis or HFA, having multiple causes of visual field defects simultaneously, and history of previous ocular surgery.

Basic data collection instrument after informed consent was filled with demographic data, ophthalmic data, disease conditions, and attached with reports of both HFA and Specvis for assessment by three senior ophthalmology consultants independently.

**Figure 1:**
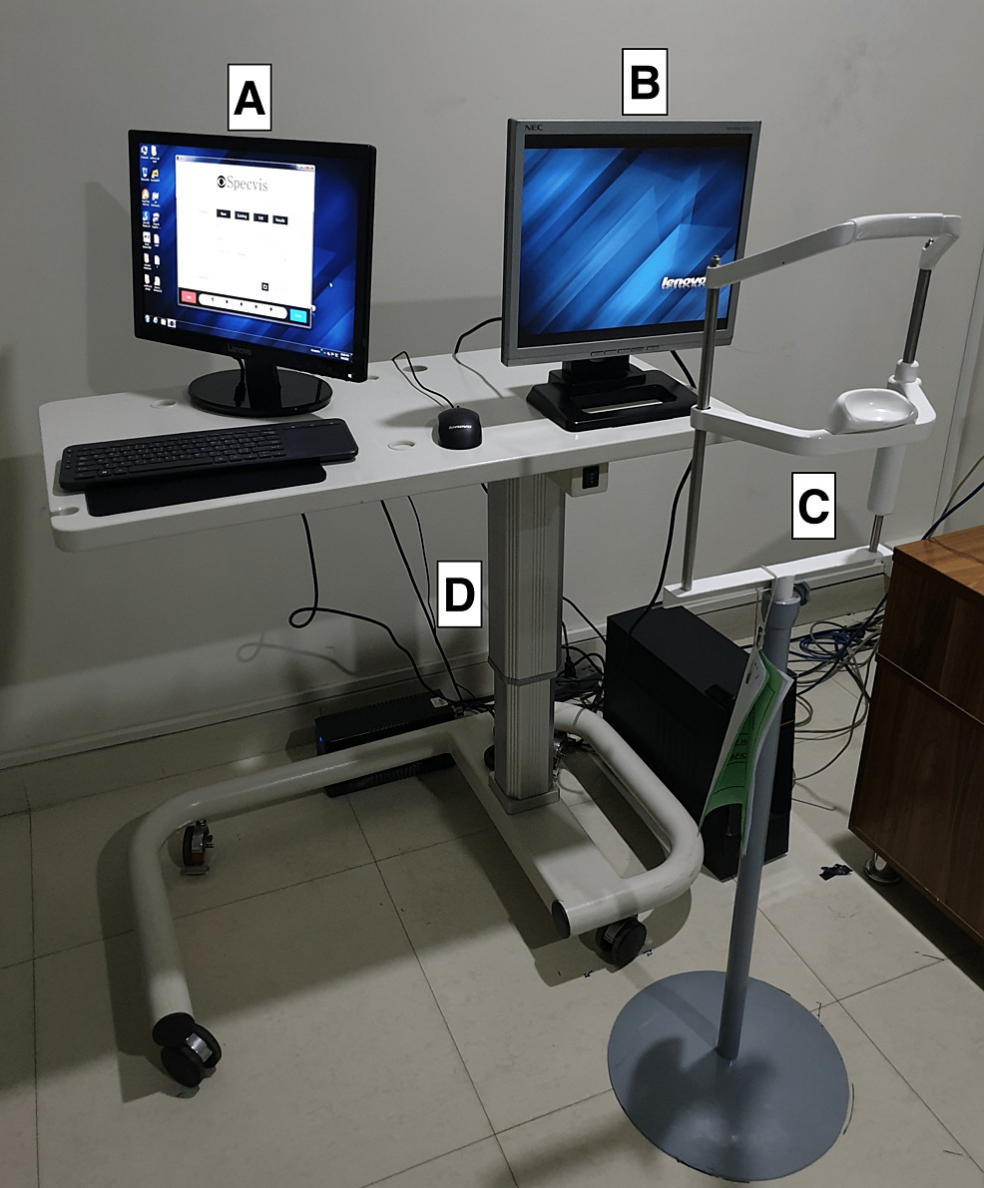
Specvis Visual Field examination setup A = Operator Screen, B = Patient Screen, C = Adjustable Chin Rest, D = Height adjustable table

**Figure 2:**
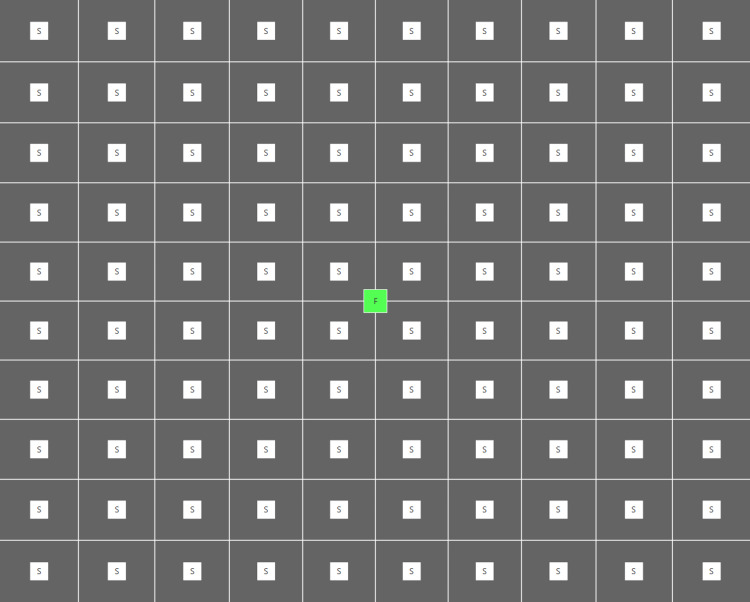
Preview of the Specvis VF stimuli distribution used in the study Screenshot from the monitor. F = Central fixation point, S = Individual stimulus points

**Figure 3:**
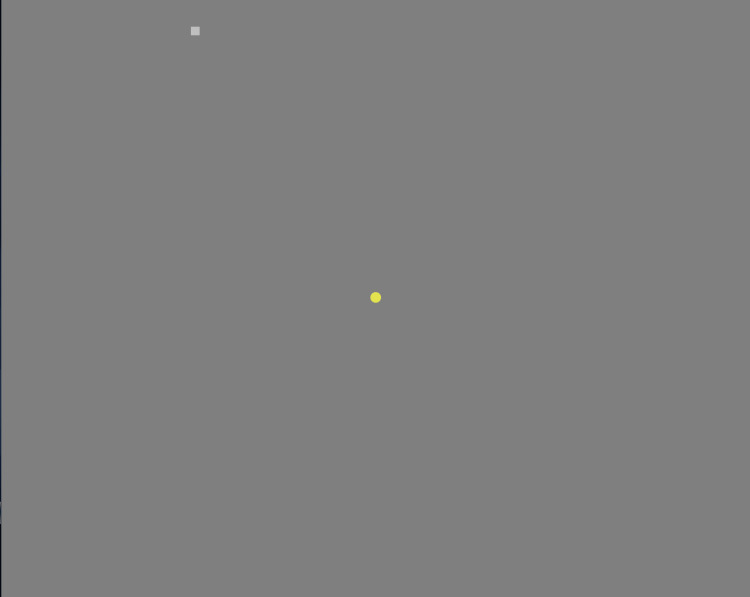
Specvis VF examination in process Screenshot from the monitor. The central small yellow circle is the fixation target, while small gray square in the upper left part of the figure is the stimulus being given at the time of this screenshot.

**Figure 4:**
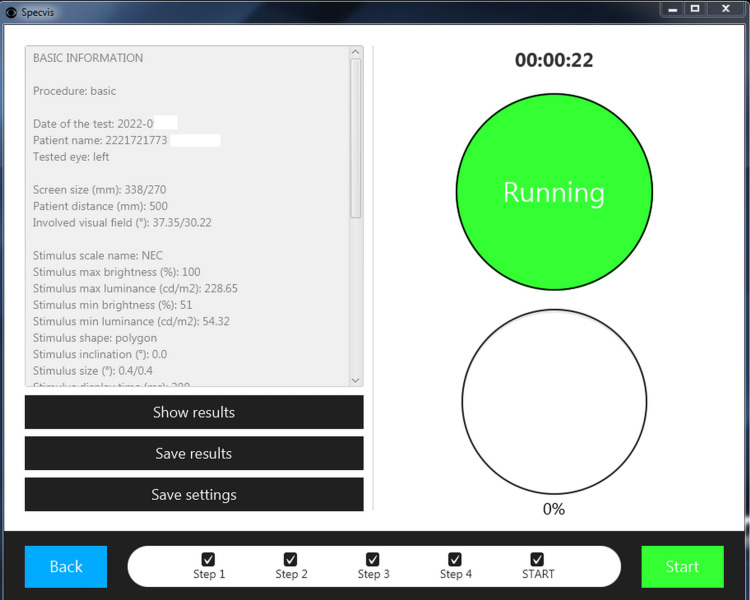
Specvis Operator window Screenshot from the operator monitor showing information on the running visual field exam.

This was a pilot study at our institute and a total of 218 eyes of 109 subjects were included in the study. Of these, 170 were healthy eyes, while 48 were diseased eyes. SAP was done on the VF 30-2 program with SITA-Fast strategy using Humphrey Field Analyzer 3 (Carl Zeiss Meditec AG, Jena, Germany). The same patient would then perform the visual field assessment on a PC with Specvis installed (Figure 1). The latest version (v1.1.1) of the software available at the start of the study was used throughout the study on the Windows (Microsoft Corp., Redmond, WA, USA) version 10 operating system with settings adjusted to match the VF 30-2 program of HFA as closely as possible (Figure 2, 3). Keeping in mind the cost-effectiveness aspect of the study, a mid-tier 17-inch PC monitor (AccuSync LCD73v by NEC Display Solutions, Tokyo, Japan) was used in the study. The screen brightness output was measured by a Lux meter (Model UT-383 by Uni-trend Technology, Guangdong, China) and entered into the software in order to obtain a more accurate visual field (VF) by the software as recommended by its author. A chinrest with appropriate distance (500mm in our setup), height and orientation (on eye level in line with fixation target) from the PC screen was set and marked in order to confer uniformity to the VF performed. On the HFA, the patient’s response was recorded by the press of a specially designed handheld button provided by the manufacturer, while on Specvis, the patient pressed the space button on a provided wireless keyboard to record their response. All the VFs were performed by a single operator to minimize the chances of inter-operator variability. A second PC monitor was also connected and used by the operator (Figure 4). Any VF with more than 7% false-positives or false-negative answers was discarded. A maximum of three attempts were allowed for each subject to understand how to perform the test and subsequently improve the reliability indices of the test performed.

**Figure 5:**
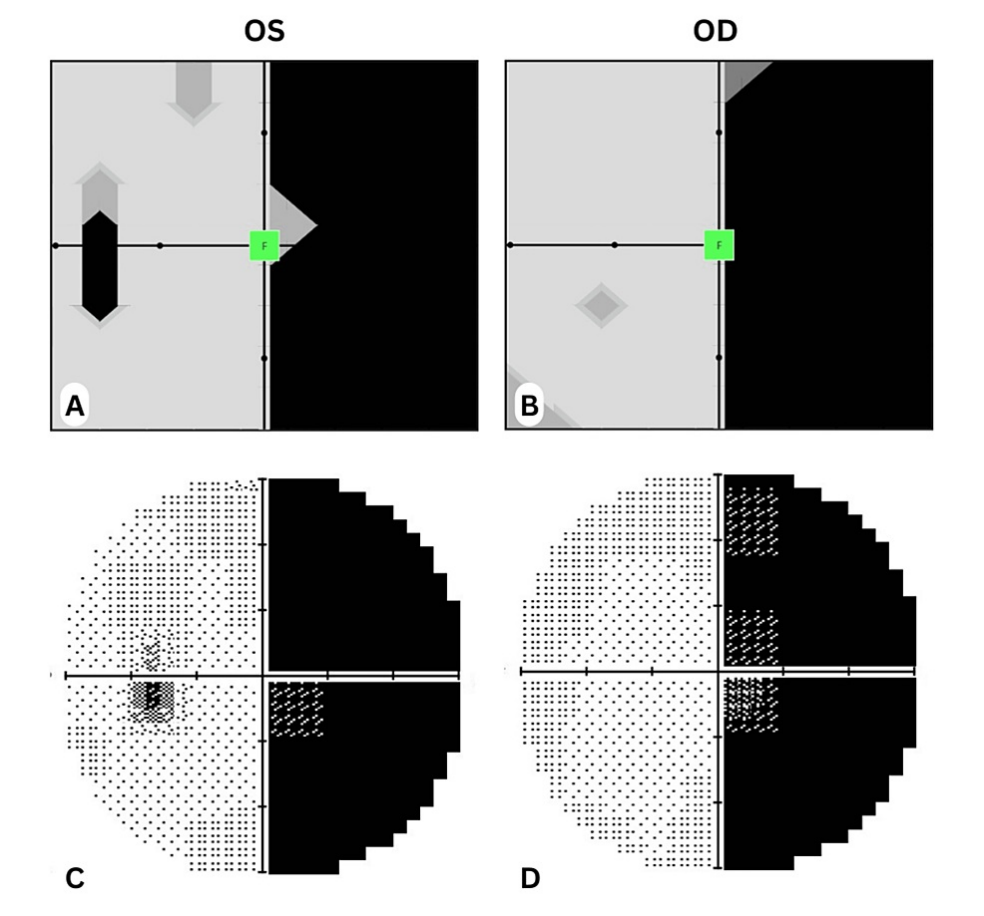
Comparison of visual field graphical maps output by Specvis and Humphrey Field Analyzer (HFA) A,B are outputs of left and right visual fields respectively as examined by the Specvis software. C,D are outputs of the left and right visual fields respectively of the same patient as examined by HFA on VF 30-2 program. The patient has a right homonymous hemianopia.

Visual fields of a subject obtained from HFA and Specvis were then coupled and sent to three different senior ophthalmologists for an independent assessment who were blinded from each other. The assessment was done by comparing the visual fields printouts in the graphical domain and scored based on a 5-point Likert scale (1 = Inadequate, 2 = Poor, 3 = Acceptable, 4 = Good, 5 = Excellent) which were then entered as ordinal data and analyzed for inter-observer reliability (Figure 5).

After each test, all subjects were also asked to rate the difficulty level of performing the test on HFA and Specvis based on a 5-point Likert scale (1 = Very difficult, 2 =Difficult, 3 = Neutral, 4 = Easy, 5 = Very Easy).

The duration of the test performed on HFA and Specvis was also noted for comparison. Statistical analysis was done using SPSS version 23 software for Windows (IBM, Armonk, NY, USA) and Intellectus Statistics (online computer software) [17].

Descriptive statistics are presented in frequencies. A Spearman correlation analysis was conducted among Score by Observer 1, Score by Observer 2, and Score by Observer 3. This was also applied to ease of using HFA vs ease of using Specvis by participants, and age. Cohen's standard was used to evaluate the strength of the relationships, where coefficients between .10 and .29 represent a small effect size, coefficients between .30 and .49 represent a moderate effect size, and coefficients above .50 indicate a large effect size [18].

## Results

Out of our total study participants, 128 (58.72%) were male and 90 (41.28%) were female. The majority of the participants were non-diseased (n = 170, 77.98%) while advanced glaucoma was the commonest disease in the diseased group (n = 22, 10.09%). The most frequently observed category of Score by Observer 1 was excellent (n = 171, 78.44%), of Score by Observer 2 was excellent (n = 107, 49.08%), and of Score by observer 3 was excellent (n = 188, 86.24%). For ease of using HFA and Specvis by participants, "Easy" was the commonest response from participants (n = 176, 80.73% and n = 159, 72.94% respectively). Frequencies and percentages are presented in Table 1.

**Table 1:** Frequency table for variables HFA: Humphrey Field Analyzer

Variable	n	%
Gender		
Male	128	58.72
Female	90	41.28
Ocular Disease		
Non-diseased	170	77.98
Mild-Moderate Glaucoma	8	3.67
Advanced Glaucoma	22	10.09
Neurological Field	10	4.59
Retinal Disease	8	3.67
Score from Observer 1		
Poor	3	1.38
Acceptable	6	2.75
Good	38	17.43
Excellent	171	78.44
Score from Observer 2		
Poor	2	0.92
Acceptable	7	3.21
Good	102	46.79
Excellent	107	49.08
Score from Observer 3		
Poor	4	1.83
Acceptable	5	2.29
Good	21	9.63
Excellent	188	86.24
Ease of using HFA by participants		
Neutral	6	2.75
Easy	176	80.73
Very Easy	36	16.51
Ease of using Specvis by participants		
Neutral	4	1.83
Easy	159	72.94
Very Easy	55	25.23
Note. Due to rounding errors, percentages may not be equal to 100%.		

The observations for age had an average of 40.71 (SD = 15.24, Min = 15.00, Max = 66.00 ). The observations for HFA duration had an average of 213.33 seconds (SD = 33.49, Min = 174.00, Max = 314.00). The observations for Specvis duration had an average of 267.36 seconds (SD = 35.98, Min = 228.00, Max = 370.00).

A significant positive correlation was observed between score 1 and score 2, with a correlation of .36, indicating a moderate effect size (p < .001, 95.00% CI = [.23, .47]). A significant positive correlation was also observed between score 1 and score 3, with a correlation of .55, indicating a large effect size (p < .001, 95.00% CI = [.45, .63]). A significant positive correlation was also observed between score 2 and score 3, with a correlation of .39, indicating a moderate effect size (p < .001, 95.00% CI = [.27, .49]). Table 2 presents the results of the correlations.

**Table 2:** Spearman correlation results among Score 1, Score 2, and Score 3

Combination	R	95.00% CI	n	p
Score1-Score2	.36	[.23, .47]	218	< .001
Score1-Score3	.55	[.45, .63]	218	< .001
Score2-Score3	.39	[.27, .49]	218	< .001

A significant negative correlation was observed between ease of using HFA by participants and age, with a correlation of -.28, indicating a small effect size (p < .001, 95.00% CI = [-.39, -.15]). This suggests that as age increases, HFA ease tends to decrease. A significant negative correlation was observed between ease of using Specvis by participants and age, with a correlation of -.26, indicating a small effect size (p < .001, 95.00% CI = [-.38, -.13]). This suggests that as age increases, Specvis ease tends to decrease. Table 3 presents the results of the correlations.

**Table 3:** Spearman correlation results among HFA Ease, Specvis Ease, and Age HFA: Humphrey Field Analyzer

Combination	r	95.00% CI	n	p
HFA Ease - Age	-.28	[-.39, -.15]	218	< .001
Specvis Ease - Age	-.26	[-.38, -.13]	218	< .001

**Figure 6:**
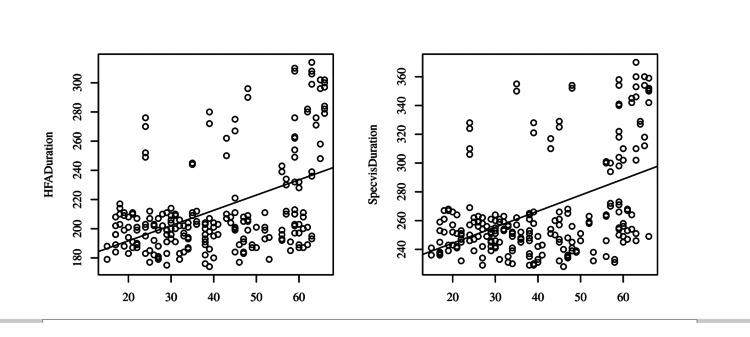
Scatterplots with the regression line added for Age and HFA Duration (left), Age and Specvis Duration (right) HFA: Humphrey Field Analyzer

A significant positive correlation was observed between age and HFA duration, with a correlation of .48, indicating a moderate effect size (p < .001, 95.00% CI = [.37, .57]). This suggests that as age increases, HFA duration tends to increase. A significant positive correlation was also observed between age and Specvis duration, with a correlation of .47, indicating a moderate effect size (p < .001, 95.00% CI = [.36, .57]). This suggests that as age increases, Specvis duration tends to increase as shown in Figure 6.

## Discussion

The modern-day glaucoma definition largely depends on optic nerve head changes and corresponding perimetric findings along with intraocular pressure as the main modifiable risk factor. As it’s a chronic and progressive optic neuropathy, diagnosis of the disease as well as progression is documented with the help of visual fields and optic disc/retinal nerve fiber layer changes. Thus, periodic visual field assessment which is reliable and reproducible is invaluable in the management of glaucoma, for which SAP is the current gold standard [19]. We have correlated Specvis, a free computer-based software for visual field analysis, with HFA. Our study was conducted during the second peak of the COVID-19 pandemic in the country when virtually all sorts of chronic patients were neglected due to limited hospital appointments and smart lockdowns [20]. The pandemic had a financial burden on the patients as well, which resulted in poor medical care for most of the patients suffering from chronic diseases requiring lifelong surveillance, including glaucoma. In the past three years, there has been a paradigm shift towards a more remote health delivery system and personal disease surveillance modalities. A number of devices and applications have been developed for home use including iCare® HOME tonometer (Icare Finland Oy, Vantaa, Finland) and electronic tablet and personal computer-based visual field analyzers [9]. Due to their very recent popularity, limited literature is available as is the case with the software (Specvis) we used in our study. To name a few such devices and applications, one developed by students at the University of California San Francisco School of Medicine in collaboration with Vivid Vision® developed a software to be operated on Virtual Reality goggles. The authors reported a very good test-retest variability and quite strong point-wise correlation and global mean sensitivity between VVP-10 schedule and HFA. However, the program is not commercially available yet and is a relatively expensive potential alternative at present [21]. Another such software used on iPad (Apple Inc., Cupertino, CA, USA) is the Visual Fields Easy app which has shown promising results in detecting moderate and advanced field defects as published by the authors in the initial validation study [22]. MRF (GLANCE Optical Pty Ltd, Melbourne, Australia) is another application developed on the iPad tablet platform. As iPad tablets are portable and widely in use, it allows VF testing outside of the clinic and in remote areas [23]. Our study results are promising and the first validation study of Specvis software. Three experienced ophthalmologists compared the grayscale results of Specvis with the results of HFA VF 30-2 of the same participant and found the comparability to be between good and excellent in more than 80% of results. The mean test duration for Specvis was slightly higher than HFA VF 30-2 using the SITA-Fast protocol i.e. 267.36 seconds ± 35.98 vs 213.33 seconds ± 33.49. Age also had a significant positive correlation with the test duration of both Specvics and HFA. The patient’s responses for ease of use of HFA and Specvis were also comparable as on the Likert scale "easy" was the most frequent response by the participants for both tests i.e. 176 vs 159 individuals respectively.

There were certain limitations to our study. Fixation monitor was not used (on Specvis or HFA) in our study as Specvis appeared to become unstable while the setting was used. Our study was also limited to just comparing the visual fields in the graphical domain due to the current nature of the result output format of Specvis. Validation based on a Likert scale may be considered a limitation in some aspects, but was likely the only feasible method to carry out such validation and was designed to be offset as much as possible through assessment by three separate ophthalmologists. A limitation of Specvis in its current state is that the points tested can be made approximately similar to different SAP patterns (e.g., 30-2, 60-4, etc), but require separate settings to be made manually as we did in our study.

Strengths of our study include a relatively large sample size and methodology of assessment and validation of results by three independent ophthalmologists.

Overall, with appropriate setup and settings as detailed by the software authors in their article along with basic usage training, this software may be able to help bridge the gap between confrontation perimetry and SAP [11]. The significantly less setup and maintenance cost of a PC as well as its wide availability when compared to specially designed SAP machines cannot be emphasized enough. The communication with the software author, Dzwiniel P, revealed that although the development of the software has been slow, it is in process of planned updates and bug fixing, which will surely improve the functionality and ease of use of the software helping it to perform better in every aspect. The open-source nature of the software also makes it amenable to be updated and modified by interested individuals and organizations other than the original software authors as well. This can very well translate into even speedier and greater improvements in the software which may not even have been envisioned earlier.

## Conclusions

Visual field examination plays a pivotal role in the diagnosis of key neuro-ophthalmic conditions. Management of glaucoma on the other hand principally requires serial visual field examinations. SAP, although the gold standard, has a significant financial burden on the healthcare systems as well as on patients’ pockets. Furthermore, situations like the recent pandemic can severely affect health delivery systems. Specvis, a computer-based free open-source software used in our study, can give promising results in identification as well as monitoring the progression of visual field defects. It may act as a significantly cost-effective alternative to SAP and should be able to bridge between visual field examination by confrontation method and SAP.
